# Transcriptional Analysis Reveals Key Genes in the Pathogenesis of Nifedipine-Induced Gingival Overgrowth

**DOI:** 10.1155/2020/6128341

**Published:** 2020-05-13

**Authors:** Yanqin Ju, Lijuan Huang, Shuwei Wang, Shouliang Zhao

**Affiliations:** ^1^Department of Stomatology, Huashan Hospital, Fudan University, Shanghai 200040, China; ^2^Shanghai Jiading District Dental Center, Shanghai 201800, China

## Abstract

**Background:**

Nifedipine-induced gingival overgrowth (NGO) is a multifactorial pathogenesis with increased extracellular matrix including collagen and glycans, inflammatory cytokines, and phenotype changes of fibroblasts. However, the molecular etiology of NGO is not well understood. The objective of this study is to investigate the key genes in the pathogenesis of NGO.

**Methods:**

In this study, we examined the proliferation and migration abilities of fibroblasts derived from patients with chronic periodontitis, nifedipine nonresponder gingival overgrowth, gingival overgrowth caused by nifedipine, and healthy normal gingiva. We conducted RNA-Seq on these four groups of fibroblasts and analysed the differentially expressed genes (DEGs).

**Results:**

Fibroblasts derived from NGO patients had higher proliferation and migration abilities than those of the other groups. Protein-protein interaction network analysis indicated that TGFB2, ITGA8, ITGA11, FGF5, PLA2G4D, PLA2G2F, PTGS1, CSF1, LPAR1, CCL3, and NKX3-1 are involved in the development of NGO. These factors are related to the arachidonic acid metabolism and PI3K/AKT signaling pathways.

**Conclusion:**

Transcriptional gene expression analysis identified a number of DEGs that might be functionally related to gingival overgrowth induced by nifedipine. Our study provides important information on the molecular mechanism underlying nifedipine-induced gingival overgrowth.

## 1. Introduction

Drug-induced gingival overgrowth (DIGO)/hyperplasia is a frequent adverse effect observed in patients treated with anticonvulsant, immunosuppressant, and some antihypertensive medications [1–3]. Nifedipine is widely used to treat hypertension and/or angina. Nifedipine-induced gingival overgrowth (NGO) is reported to be the most frequent form of DIGO, with an incidence ranging from 6.3% to 83% [4–6]. However, the molecular etiology of NGO is not well understood.

Several possible mechanisms and pathways of NGO have been proposed over the years. Several factors are thought to influence the relationship between nifedipine and gingival overgrowth, including local inflammation, drug-induced alterations in gingival connective tissue homeostasis, and genetic predisposition [7, 8]. NGO is characterized by cell proliferation and extracellular matrix (ECM) component accumulation in gingival connective tissues. Inflammatory cytokines, such as interleukin-1*β* [9] and interleukin-6 [10], have also been reported to synergistically enhance proliferation and collagen production in human gingival fibroblasts (hGFs) [11]. In addition, collagen synthesis is controlled by matrix metalloproteinases (MMPs) and tissue inhibitor of metalloproteinases (TIMPs). MMPs are key enzymes involved in the degradation of collagen and ECM components during periodontal inflammation and NGO [12, 13]. Moreover, not all patients taking nifedipine develop gingival overgrowth. In the literature, patients who show NGO are termed “responders,” and those who do not are termed “nonresponders.” Such interindividual susceptibility to gingival changes may be related to genetic predisposition, which can influence a variety of factors in the drug-cell-plaque-induced inflammation interaction [14]. Our previous study has proved that MMP-2 and MMP-9 played significant roles in regulating nifedipine-induced gingival overgrowth development and progression. Nifedipine treatment accompanied by local inflammation regulated MMP-2 and MMP-9 expression, which was most likely associated with NGO severity [13]. Therefore, we strongly speculate that changes in critical genes or effector molecules play a significant role in the pathogenesis of NGO.

## 2. Materials and Methods

### 2.1. Experiment Groups and Tissue Preparation

In our study, gingival specimens were obtained from 12 patients ranging from 40 to 70 years old (*n* = 3 for each group). Inclusion criteria were as follows: (1) the average time of taking nifedipine for high blood pressure was 3-8 years; (2) all patients had no other systemic diseases or history of taking antibiotics within 3 months before enrollment; (3) women were not pregnant and did not take contraceptives; (4) the number of teeth reserved in the mouth ≥ 20; and (5) informed consent was signed for treatment research. Objectives in the NNGO group and NGO group should meet items 1-5 at the same time, and objectives in the control group and CP group should meet items 2-5. In addition, the diagnosis of periodontitis was based on the following: (1) interdental clinical attachment loss (CAL) is detectable at ≥2 nonadjacent teeth, or (2) buccal or oral CAL ≥ 3 mm with pocket probing depth (PPD) ≥ 3 mm is detectable at ≥2 teeth but the observed CAL cannot be ascribed to nonperiodontitis-related causes [15]. Exclusion criteria were as follows: (1) patients with gingival hyperplasia due to other drugs (such as phenytoin sodium and cyclosporine A); (2) patients with other systemic serious diseases; and (3) patients using hormone therapy in the past 3 months.

Cases were divided into four groups, and the status of periodontal disease activity was measured and assessed by gingival index (GI), gingival margin level (GML), and CAL. Periodontal manual probe and measurements were recorded in 28 teeth at four sites, namely, distobuccal, midbuccal, mesiobuccal, and lingual, per tooth three times. Each tooth was scored as the average of 4 dental scores. The scoring criteria of GI score [16] are as follows: 0 points: healthy gingivae; 1 point: mild inflammation of the gingivae, slight changes in gingiva color, mild edema, and no bleeding during exploration; 2 points: moderate inflammation of the gingivae, reddish gingivae, bright edema, and bleeding diagnosis; and 3 points: severe inflammation of the gingivae, obvious swelling or ulceration of the gingivae, and a tendency to bleed automatically. CAL means the distance from the cementoenamel junction to the bottom of the periodontal pocket which indicates the degree of attachment loss. GML means the distance from the gingival margin to the cementoenamel junction which indicates the degree of gingiva degradation.

All the patients were made to undergo oral ultrasonic scaling to remove deposits which might interfere in probing pockets and detecting CAL.

(Group A) Patients with periodontally healthy tissues (GI score = 0, GML = 0 mm, and CAL = 0 mm) who needed the third molar extracted (NOR)
(Group B) Patients with severe chronic periodontitis (GI score = 2, GML = 3 mm, and CAL ≥ 5 mm) who needed the third molar extracted (CP)
(Group C) Patients with nifedipine non-responder gingival enlargement (GI score = 1, GML = 0 mm, and CAL = 0 mm) who needed the third molar extracted (NNGO)
(Group D) Patients with gingival enlargement (GI score = 1, GML = −3 mm, and CAL = 0 mm) caused by nifedipine who needed periodontal treatment (NGO)

Informed consent was obtained from each participant under a protocol approved by the Ethics Committee of Fudan University. Tissues from the proximal papillae of the mandibular third molars were made into paraffin sections, and one representative section from each specimen was stained with hematoxylin and eosin (H&E), while the remaining gingival tissue was used to culture fibroblasts for RNA-Seq. The samples were fixed in 10% buffered formalin for 48 h at 4°C for further histological examination.

### 2.2. Cell Culture

Briefly, after isolation of the gingival tissue, gingival epithelial tissue was eliminated in the culture dish with a small amount of Dulbecco's modified Eagle's medium (DMEM) containing 10% fetal bovine serum (FBS). The tissue was cut into 1 mm^3^ blocks using ophthalmic scissors, transferred into culture flasks and cultured in an incubator at 37°C, 5% CO_2_, and 95% relative humidity. The medium was changed daily. The cells were grown to semiconfluence, harvested by trypsinization at 37°C for 2 min, and then subcultured in a new dish. The experiments were performed using fourth-passage fibroblasts.

### 2.3. CCK8 Assays

The Cell Counting Kit 8 (CCK8, Dojindo, Kumamoto, Japan) was used to assess hGF proliferation. According to the manufacturer's instructions, 1.5 × 10^3^ cells/ml were seeded into 96-well culture plates. The growth medium was replaced with serum-free DMEM after cells were allowed to attach for 24 h. Then, 10 *μ*l of CCK-8 solution (5 g/l) was added and incubated for a further 2 h. A Multiskan GO spectrophotometer (Thermo Scientific, Waltham, MA, USA) was used to measure absorbance at 450 nm.

### 2.4. Transwell Cell Migration Assay

Cell motility was determined in vitro using a Transwell chamber (Costar, Corning, NY, USA). The cells were trypsinized and placed into the upper wells of the Transwell chamber (40,000 cells per well) in 100 *μ*l of serum-free DMEM. In the lower section of the chamber, 600 *μ*l of DMEM containing 10% FBS was added. The cells were then cultured in an incubator for 24 h. After the nonmigrated cells were scraped off, the membranes were fixed with methanol, and cells were counted after staining with 4′, 6-diamidino-2-phenylindole (DAPI; KeyGEN BioTECH, Nanjing, China). The cells in five separate fields were counted using light microscopy at 200x magnification.

### 2.5. RNA-Seq and Protein-Protein Interaction Network Analysis

To construct each cDNA library, total cellular RNA was extracted from fibroblasts using Trizol reagent (Invitrogen, Carlsbad, CA, USA). Each cDNA library was constructed according to the manufacturer's guidelines, and next-generation sequencing was carried out in the company (WuXi AppTec Co. Ltd, Wuxi, China), using an Illumina HiSeq 2000 platform (IGA, Udine, Italy). RNA-Seq compares the number of reads that align to a specific transcript in different samples or cDNA libraries. Expression was calculated using RPKM (Reads Per Kilobase of exon model per Million mapped reads) normalization, while accounting for the length of the transcript, the number of base pairs, and the total number of reads in the transcriptome [17]. This normalization eliminates the influence of different gene lengths and sequencing levels on the calculation of gene expression. Genes were considered differentially expressed if they exhibited a Benjamini and Hochberg-adjusted *p* value (FDR) of 5%, and a mean fold change of 0.2. Bioinformatics analysis of DEGs was based on RNA-Seq results. Moreover, to further study the relationships among DEGs at the protein level, we generated a PPI network (based on the identified DEGs) by integrating protein information from the Search Tool for the Retrieval of Interacting Genes (STRING) database (http://string-db.org/). The Database for Annotation, Visualization, and Integrated Discovery (DAVID) online platform (https://david.ncifcrf.gov/) was used to perform Gene Ontology (GO) analysis of the DEGs.

### 2.6. Statistical Analysis

Statistical calculations were carried out using SPSS version 20 (IBM Corp., Armonk, NY, USA). Student's *t*-test or one-way analysis of variance (ANOVA) was performed to determine statistical significance for each comparison; *p* < 0.05 was considered statistically significant.

## 3. Results

### 3.1. The Clinical Features

**Figure 1:**
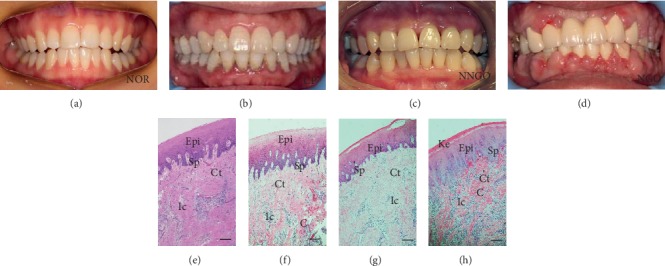
Photographs of mouths and representative H&E staining of the gingival tissues showing the four experimental groups (NOR: normal; CP: chronic periodontitis; NNGO: nifedipine nonresponder gingival overgrowth; NGO: nifedipine-induced gingival overgrowth). Epi: squamous epithelium; Ct: connective tissue; Ic: inflammatory cell; Sp: spinous cell layer; Ke: hyperorthokeratosis; C: collagen bundles. Bar = 100 *μ*m.

The normal gingivae were pale pink and glossy. The margin was knife edged and scalloped. A streamlined papilla was often grooved by a sluiceway approximately 1-2 mm depth, and the attached gingivae were stippled. The gingiva was firm, including multiple blood vessels and nerves (Figure 1(a)). In the CP group, the knife-edged margin became rounded, the interdental sluiceway was lost, and the gingival surface became smooth and glossy. Gingival bleeding occurred frequently upon touching (Figure 1(b)). The clinical features of gingivae in the NNGO group were similar to those of normal gingivae (Figure 1(c)). In the NGO group, enlargement was either localized or generalized, affecting the entire mouth. The entire papillae and surrounding tissues were enlarged, giving the gingival tissues a lobulated appearance (Figure 1(d)).

### 3.2. Histological Findings

In the NOR group, gingival tissue exhibited the structure of stratified squamous epithelium (EPi), with short, organized epithelium spikes. Approximately 3 to 4 layers of acanthocytes were present, and granule cells were flat, with well-adhered keratin. Cuboidal basal cells with hyperchromatic basophilic nucleus were perpendicular to the basement membrane. The lamina propria had a large amount of connective tissue (Ct), which contained collagen fiber bundles and fibroblasts, scattered with some chronic inflammatory cells (Ic) and lymphocytes (Figure 1(e)). However, each layer of epithelial cells in NGO was increased, and the keratinized epithelium was thicker. The excessive epithelial spikes were wider, with irregular elongation, and some even crossed the reticulate thickening of the prickle cell layer. The spinous epithelial cells were also larger, and collagen bundles appeared wavy. The connective tissue collagen fibers were increased in the lamina propria. The fibroblasts were plump, and chronic inflammatory cells were more frequently observed than in the NOR group (Figure 1(h)). In the CP group, the acanthocyte layer was thickened, epithelium spikes were elongated, and connective tissue was increased, with more inflammatory cell infiltration (Figure 1(f)). Simultaneously, the microscopic appearance of tissue from the NNGO group was not clearly distinguishable from that of the NOR group (Figure 1(g)).

### 3.3. hGFs Derived from NGO Patients Had High Abilities of Proliferation and Migration

**Figure 2:**
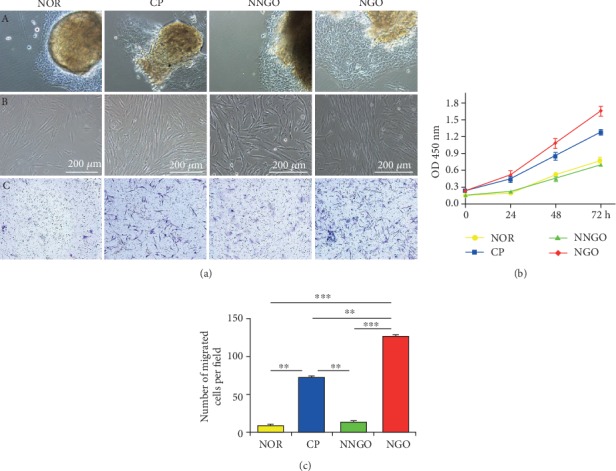
hGFs derived from NGO patients had high abilities of proliferation and migration. (a) The gingival fibroblasts of four groups were cultured. The proliferation (b) and migration (c) ability of the cells was detected by CCK8 and Transwell assay. NOR: normal; CP: chronic periodontitis; NNGO: nifedipine nonresponder gingival overgrowth; NGO: nifedipine-induced gingival overgrowth. ^∗^*p* < 0.05, ^∗∗^*p* < 0.01, and ^∗∗∗^*p* < 0.001.

hGFs obtained after digestion from the four groups presented elongated, spindle-shaped adherent growth (Figure 2(a), A and B). CCK8 assays were performed to investigate hGF proliferation. hGFs derived from the NGO and CP groups showed significantly enhanced proliferation comparing with those from the NOR and NNGO groups (*p* < 0.05). However, hGFs derived from the NGO group exhibited higher proliferation compared with those from the CP group (*p* < 0.05) (Figures 2(a), B and 2(b)). The migration rates of hGFs were subsequently measured using Transwell chambers. hGFs derived from the NGO group were more capable of migration compared with those from the CP group (Figures 2(a), C and 2(c)).

### 3.4. Transcriptomic Analysis of hGFs Derived from Nifedipine-Induced Gingival Overgrowth

**Figure 3:**
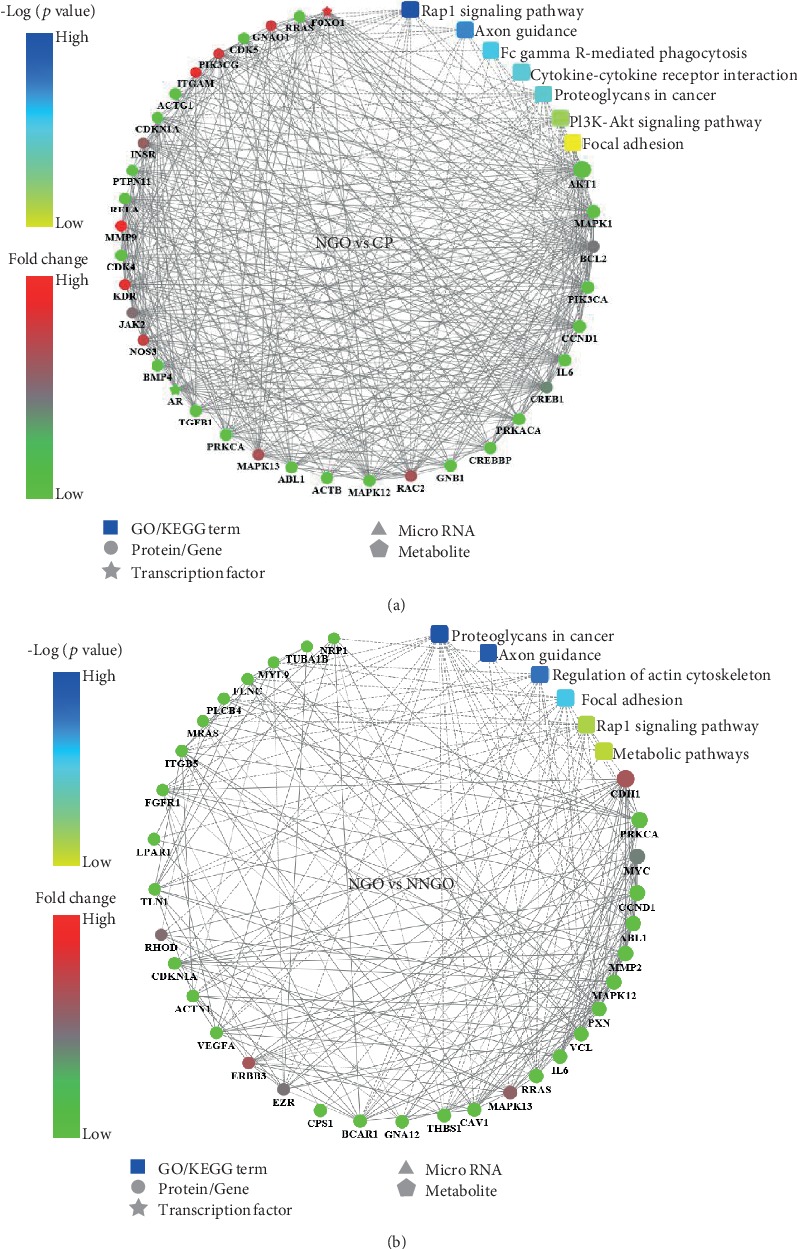
Protein–protein interaction network of the differentially expressed genes (DEGs).

To assess differences in hGF gene expression between NGO and the other groups, we used a volcano plot to identify fold changes and statistical significance (Figure S2A). First, 4040 (1866 upregulated and 2174 downregulated), 2867 (1141 upregulated and 1726 downregulated), and 11032 (6720 upregulated and 4312 downregulated) genes were identified in the NGO group that displayed fold changes > 2 (*p* < 0.05) versus the other three groups. Therefore, these genes were identified as differentially expressed genes (DEGs). PPI analysis revealed several DEGs in the NGO group compared with those in the CP group, including NOS3, ITGAM, RAC2, PIK3CG, MMP9, TGFB1, and IL6 (Figure 3(a)). GO analysis demonstrated that NGO might be related to the cytoplasm, cytoplasmic part, protein binding, and binding functions (Figure S1A). KEGG analysis indicated that these DEGs might be involved in the Rap1 signaling pathway, the PI3K-Akt signaling pathway, and axon guidance (Figures S1B and S1E).

To explore the different susceptibilities to nifedipine, we analysed the DEGs between the NGO and NNGO groups and identified several key DEGs, including FGFR1, VEGFA, IL6, MMP2, CDH1, ERBB3, RHOD, and MAPK13. GO analysis revealed that NGO might be related to the cytoplasm, cytoplasmic part, binding, and protein binding (Figure 3(b)). KEGG results showed that these DEGs are involved in proteoglycans in cancer, regulation of actin cytoskeleton, and metabolic pathways (Figure S1F).

**Figure 4:**
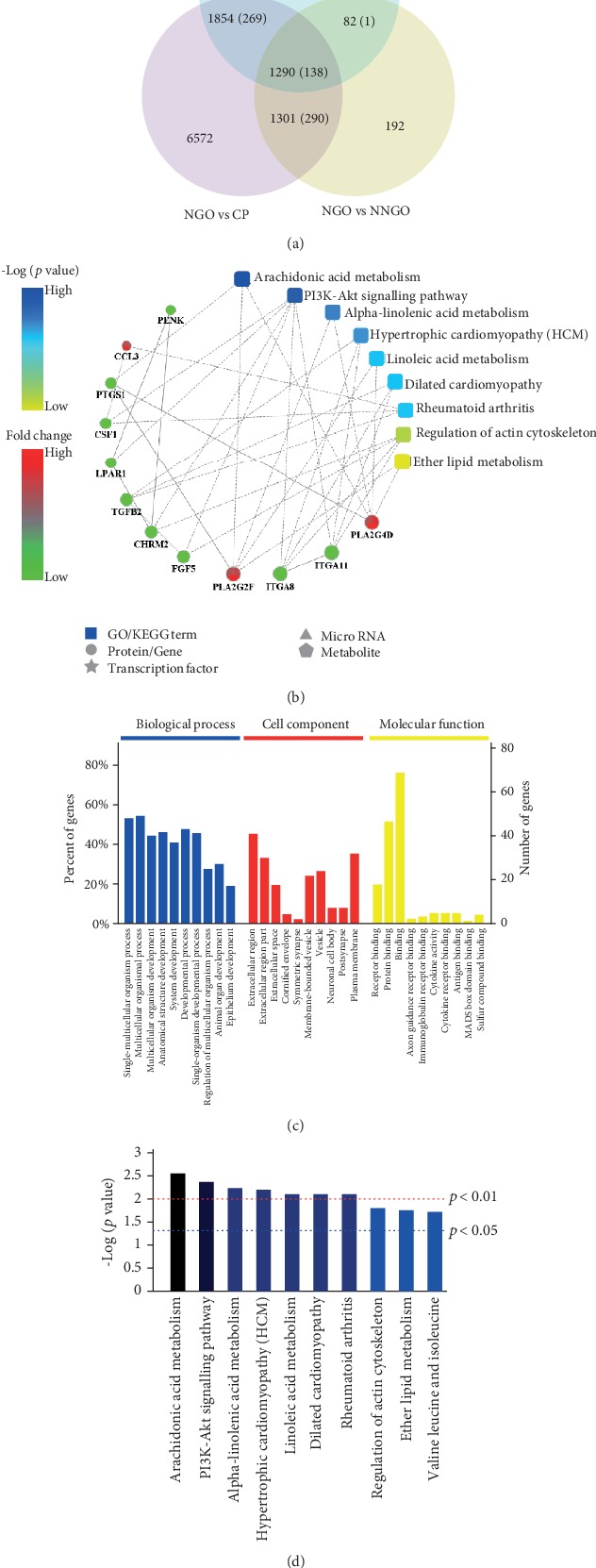
RNA-Seq analysis. (a) The distributions of the DEGs and their overlapping expression in different groups were illustrated by Venn diagram analysis. (b) Protein–protein interaction network of the differentially expressed genes (DEGs). The GO analysis (c) and Kyoto Encyclopedia of Genes and Genomes (KEGG) analysis (d) of these genes; *x*-axis is the function description corresponding to the KEGG signal pathway. *y*-axis is the *p* value; *p* value < 0.05 means significant enrichment. NOR: normal; CP: chronic periodontitis; NNGO: nifedipine nonresponder gingival overgrowth; NGO: nifedipine-induced gingival overgrowth.

Finally, the distributions of the DEGs and their overlapping expression in these different groups are illustrated by a Venn diagram in Figure 4(a), which shows that 138 DEGs were altered in NGO compared with those in the other groups, indicating that these 138 genes may be affected by nifedipine (Table S1). Based on PPI network analysis, we discovered several significant DEGs that may participate in the regulation of NGO, such as TGFB2, ITGA8, ITGA11, FGF5, PLA2G4D, PLA2G2F, PTGS1, CSF1, LPAR1, CCL3, and NKX3-1 (Figure 4(b)). These factors might be involved in PI3K-AKT signaling (Figure 4(d)). In addition, GO analysis showed that NGO was closely related to cell response to stimulus and to protein binding (Figure 4(c)).

## 4. Discussion

Nifedipine is the most frequently implicated calcium channel antagonist in DIGO [18–20], yet the underlying molecular mechanism remains unclear. Currently, some of the most cited causes and risk factors of gingival overgrowth include gender, genetics, duration of administration, and inflammation [13, 14, 21]. The pathogenic mechanisms of DIGO are known to be genetically predetermined, as gingival fibroblasts are more sensitive to GO-inducing drug than other fibroblast subpopulations. Such fibroblast heterogeneity leads to variations in the production of potentially proliferative, fibroblastic cytokines/GFs and the environmental response related to ECM components [22]. In the present study, we cultured and analysed hGFs derived from NGO patients and observed that these hGFs exhibited enhanced migration and proliferation abilities.

RNA-Seq provides an even more precise measurement of transcriptional levels of gene expressions involved in DGO than all other methods, enabling us to further elucidate the molecular mechanism underlying NGO. In this study, transcriptome analyses yielded rich information on gingival fibroblasts from the NGO group. Previous work has suggested that local inflammation, especially dental plaque, is a vital factor in the etiology of DIGO [23–25]. To eliminate the effect of inflammatory factors on gingival hyperplasia, fibroblasts from patients with gingival hyperplasia were compared with those from periodontitis patients. Based on PPI network analysis between the NGO and CP groups, we identified key novel DEGs in the fibroblasts derived from the NGO group including NOS3, RAC2, ITGAM, and PIK3CG, which were upregulated compared to the CP group. To our knowledge, this is the first report describing the relationship between these novel genes and NGO. NOS3 promotes the synthesis of nitric oxide (NO), an intracellular signaling molecule that regulates vasodilation via a cGMP signaling-mediated signal transduction pathway [26]. Meanwhile, RAC2 augments reactive oxygen species (ROS) production by increasing NOS activity [27], through NADPH oxidase [28]. ROS are reported to play a critical role in oxidative stress, which seems to be linked to the initiation and progression of fibrotic diseases [27, 29]. PIK3CG is a key factor of cell growth, proliferation, and motility, involved in the endothelial progenitor migration [30]. Moreover, IL6 and TGFB1 were identified as significant DEGs in the NGO group compared with the CP group. IL6 is related to immune-inflammatory features associated with NGO. TGFB1 plays a key role in fibrosis of different tissues, such as skin, liver, kidney, eye, lung, and the cardiovascular system. TGFB1 stimulates fibroblastic population and ECM deposition of fibronectin and glycosaminoglycans (GAGs) [31–33]. MMP9 was significantly upregulated in NGO fibroblasts. Our previously study illustrated that MMP9 is a potential contributor to NIGO development and is most likely associated with NIGO severity [13]. These results suggest that NGO is influenced by various factors including cytokines and enzyme, although the inflammatory process is ultimately responsible for the severity of NGO [34].

Considerable sequestration of nifedipine has been observed in patients who exhibit significant gingival changes arising from treatment with this drugs [35, 36]. However, not all patients receiving nifedipine develop gingival overgrowth. Gingival fibroblasts exhibit functional heterogeneity in response to various stimuli [7]. Comparing the NOG and NNGO groups, we identified several key DEGs such as FGFR1, VEGFA, IL6, MMP2, CDH1, ERBB3, RHOD, and MAPK13. FGF is a fibroblast mitogen molecule with the differentiation functions related to fibroblastic proliferation according to GO analysis [37]. FGFR1-ligand binding activates several signaling cascades and mediates the RAS and MAPK/ERK signaling pathways [38]. VEGFA promotes endothelial cell proliferation and differentiation, induces microvascular hyperpermeability, and participates in ECM remodeling [39]. CDH1 is involved in regulation of cell-cell adhesion, mobility, and proliferation of epithelial cells [40]. RHOD is involved in the internalization and trafficking of activated tyrosine kinase such as PDGFRB and ERBB3 [41]. PDGF stimulates IGF synthesis, which leads to increased fibroblast collagen synthesis and may be related to the stimulation of mesenchymal tissues during periodontal regeneration [42]. MMP2 was downregulated in fibroblasts derived from the NOG group compared with those from the NNGO group. Decreased MMP-2 levels may be associated with impaired tissue remodeling, leading to pathological collagen deposition and tissue fibrosis [43]. MAPK13 is one of the four p38 MAPKs that play an important role in cellular responses evoked by extracellular stimuli such as proinflammatory cytokines, the regulation of the epidermal keratinocyte differentiation, and apoptosis. Our results indicate that these DEGs are closely related to nifedipine sensitivity in fibroblasts and are involved in proteoglycans in cancer and regulation of the actin cytoskeleton. The development of NGO might be regulated by the RAS, MAPK/ERK, and RAP1 signaling pathways.

To further identify the mechanism of NGO, we analysed the overlapping gene expression between the NGO and NNGO groups, the NGO and CP groups, and the NGO and NOR groups. Based on PPI network analysis, we identified key DEGs including TGFB2, ITGA8, ITGA11, FGF5, PLA2G4D, PLA2G2F, PTGS1, CSF1, LPAR1, CCL3, and NKX3-1. TGFB2 suppresses IL-2 dependent T-cell growth. Downregulation of TGFB2 expression is associated with the reduction of cyclosporine-induced GO in rats treated with roxithromycin [44]. FGF5 plays an important role in regulating cell proliferation via ERK1/2 activation [45]. ITGA8 plays a role in organogenesis, likely by regulating the recruitment of mesenchymal cells into epithelial structures [46]. ITGA11 is a collagen receptor [47]. ITGA11 knockdown in hepatic stellate cells (liver-specific myofibroblasts) markedly reduced transforming growth factor *β*-induced differentiation and fibrotic parameters [48]. It might be involved in fibrogenic signaling. LPAR1 is a lysophosphatidic acid receptor related to actin cytoskeleton reorganization, cell migration, differentiation, and proliferation. LPAR1 activates G proteins to trigger cytoplasmic Ca^2+^ influx. LPAR1 contributes to fibrosis and cell migration in response to injury [49, 50]. CCL3 (also known as macrophage inflammatory protein-1*α*) is a member of the CC chemokine family. It is classified as a macrophage-derived inflammatory mediator [51]. PLA2 is a superfamily of enzymes that catalyzes the production of free fatty acids and lysophospholipids [52]. PLA2 regulates IL-1*β*-induced chemokine expression [53], which might be associated with the inflammation during NGO. The androgen-regulated homeodomain transcription factor NKX3-1 plays a role in early prostate development and functions as a prostate-specific tumor suppressor [54]. CSF1 promotes proinflammatory chemokine release and regulates membrane ruffling, cell adhesion, and cell migration [55]. PTGS1 mediates prostaglandin E2 (PGE2) production and promotes vascular smooth muscle cell proliferation. These factors are involved in the arachidonic acid metabolism and PI3K/AKT signaling pathways.

In conclusion, this study revealed a number of DEGs that may be functionally related to gingival overgrowth induced by nifedipine. Although our study only illustrated the role of fibroblasts in this process, it provides an important information and a certain direction for further research on the molecular mechanism underlying this condition. Nevertheless, there are some limitations of this study. The main limitations are lack of verification experiment in the cells and small sample size. Therefore, larger study groups and further study are required for clarifying these genes or signal pathway.

## Data Availability

All data used to support the findings of this study are included within the article.

## Supplementary Materials

Supplementary Materials Table S1: list of the main overlapping genes. Figure S1: (A–C) the GO analysis of DEGs in NGO group versus NOR, CP, and NNGO, respectively. (D–F) KEGG analysis of those groups. Figure S2: RNA-Seq analysis of NGO vs. other groups. (A) Volcano plot to identify the fold changes and statistical significance. (B) Protein–protein interaction network of the differentially expressed genes (DEGs).
