# Supplementary material for: Transcriptional Analysis Reveals Key Genes in the Pathogenesis of Nifedipine-Induced Gingival Overgrowth
Source: Anal Cell Pathol (Amst). 2020 May 13;2020:6128341. doi: 10.1155/2020/6128341 (PMC7242917; doi:10.1155/2020/6128341)

**Transcriptional Analysis Reveals Key Genes in the Pathogenesis of Nifedipine-induced Gingival Overgrowth**

Figure legends

Table S1. List of the main overlapping genes.

Figure S1. (A-C) The GO analysis of DEGs in NGO group *versus* NOR, CP, NNGO, respectively. (D-F) KEGG analysis of those groups.

Figure S2. RNA-seq analysis of NGO vs other groups. (A) Volcano Plot to identify the fold changes and statistical significance. (B) Protein – protein interaction network of the differentially expressed genes (DEGs).

Table S1. List of the main overlapping genes.

|  | NGO_vs_CP | | NGO_vs_NNGO | | NGO_vs_NOR | |
| --- | --- | --- | --- | --- | --- | --- |
| gene id | log2FoldChange | pvalue | log2FoldChange | pvalue | log2FoldChange | pvalue |
| RPL21P7 | -3.976160296 | 5.25E-07 | 8.129922579 | 1.88E-22 | 2.543682667 | 0.030095264 |
| LCE3A | 8.617332187 | 1.45E-09 | 8.002024466 | 4.29E-24 | 6.241789651 | 2.31E-16 |
| LCE3C | 5.039735742 | 0.004764361 | 7.19624066 | 1.19E-15 | 6.262934204 | 3.30E-13 |
| LINC01527 | 5.63977401 | 0.000708799 | 6.862741648 | 4.63E-15 | 4.106194014 | 0.000199263 |
| LAMC1 | -2.845518566 | 3.39E-19 | 6.646407706 | 2.05E-13 | 6.057984524 | 1.99E-13 |
| ZC3H7B | 2.918963659 | 0.019477038 | 5.487324042 | 3.41E-12 | 6.957762667 | 1.71E-13 |
| SPIC | 10.69541271 | 3.02E-16 | 5.247064808 | 7.46E-09 | 2.642087422 | 0.026000196 |
| VGLL1 | 7.08936479 | 3.68E-06 | 5.208529392 | 8.19E-08 | 3.707014835 | 4.79E-05 |
| IGHV4-55 | 6.523136636 | 4.01E-05 | 5.008334855 | 4.51E-09 | 6.406794279 | 7.88E-11 |
| TXNP4 | 5.015772334 | 3.21E-08 | 4.901286277 | 3.65E-08 | 3.098837997 | 0.002202884 |
| ARHE | 4.744104221 | 0.007097258 | 4.701841051 | 7.95E-09 | -0.916321381 | 0.033500186 |
| C6orf15 | 6.703192252 | 2.12E-05 | 4.693352637 | 3.61E-06 | -2.605806992 | 0.030920944 |
| RGMB | 3.677225843 | 0.008495086 | 4.647333872 | 3.98E-09 | 3.415473811 | 4.07E-05 |
| PITPNM2 | 5.466836098 | 0.001260104 | 4.640211659 | 3.39E-07 | 2.502766201 | 0.02984568 |
| KLHL38 | -4.008716358 | 5.01E-06 | 4.416325261 | 4.42E-07 | 4.132663456 | 3.47E-07 |
| MKX | -4.098233073 | 5.51E-33 | 4.3389153 | 7.57E-06 | 3.44497071 | 0.00153376 |
| IGF2BP3 | -3.333129704 | 6.83E-15 | 4.16077996 | 6.23E-06 | 2.389432309 | 0.020668324 |
| MIR137HG | -3.570501876 | 0.002129597 | 3.939269994 | 0.000184715 | 2.602337253 | 0.007173031 |
| TRPV3 | 4.30797038 | 0.000911024 | 3.91554752 | 0.000166484 | 2.178170887 | 0.011897172 |
| IGKV2-29 | 10.77600138 | 5.36E-16 | 3.896834593 | 0.000277324 | 4.722392002 | 4.12E-05 |
| IGFL2 | 6.579710211 | 2.82E-05 | 3.866662534 | 0.000218181 | 4.26335523 | 0.000102302 |
| ACOD1 | 2.259302584 | 1.18E-06 | 3.726348494 | 2.20E-09 | 3.437281769 | 0.000307857 |
| FAM196B | -2.930814663 | 0.005887833 | 3.667547665 | 0.000515058 | 3.699957407 | 2.37E-05 |
| CCL24 | 2.671384745 | 0.027519918 | 3.459660644 | 0.001404085 | 2.845770086 | 0.006140142 |
| CTXN3 | 5.98999753 | 0.000255679 | 3.426448962 | 0.000545127 | -2.96315809 | 0.001930224 |
| IGHD1-1 | 5.370497962 | 0.001512187 | 3.389679083 | 0.000454002 | 3.754006994 | 0.000133807 |
| SERPINE2 | 4.441772068 | 0.000556056 | 3.373572943 | 0.001795347 | 2.517193467 | 0.018006533 |
| SNX18 | 5.768339262 | 0.000464498 | 3.332628618 | 0.000571043 | 2.74854112 | 0.003384513 |
| RNU7-45P | 4.108363485 | 1.03E-06 | 3.31785336 | 0.000264472 | 2.311396566 | 0.002900791 |
| SLC16A3 | 5.713188741 | 0.000578943 | 3.310941968 | 0.000860832 | 4.274846044 | 0.000103971 |
| MIR3945HG | 5.900580797 | 0.000354922 | 3.306267304 | 0.000723472 | 4.380366929 | 5.85E-05 |
| CBLN1 | 6.158675912 | 0.000256061 | 3.275867736 | 0.003101406 | -2.706622055 | 0.002752931 |
| GATA6 | 2.678031401 | 0.002095435 | 3.104448545 | 0.000120471 | -2.964394238 | 0.005202397 |
| IGHD3-3 | 5.652836584 | 0.000674597 | 3.095994612 | 0.003108324 | 2.744455544 | 0.017298435 |
| TM4SF20 | 2.028739626 | 0.001774653 | 3.08736543 | 5.68E-05 | 1.749298767 | 0.011106417 |
| CIDEA | 6.219566189 | 0.000186968 | 3.074056926 | 0.003356399 | -2.810723589 | 0.001810885 |
| ACTBP11 | 6.081807739 | 0.000176636 | 3.036595654 | 0.00140423 | -3.105697019 | 0.000272004 |
| HHIPL1 | -2.760830251 | 7.40E-12 | 3.029900988 | 0.005474007 | 1.586202503 | 0.005194621 |
| PFN2 | 3.728398018 | 4.08E-05 | 3.009166848 | 0.003550045 | 2.306195385 | 0.031570177 |
| TRIM49C | 4.06199938 | 5.26E-16 | 2.907770178 | 2.09E-08 | 2.438920396 | 1.99E-07 |
| PLOD1 | -2.331344887 | 5.35E-22 | 2.869044157 | 0.002876487 | 2.674443563 | 0.002504851 |
| ZFHX4 | -4.105094518 | 0.024664969 | 2.817634641 | 1.39E-10 | 4.478460717 | 1.03E-12 |
| CARD18 | 5.817301032 | 0.000404536 | 2.796535555 | 0.003464182 | 2.593160135 | 0.030180322 |
| IGLC5 | 4.191986526 | 0.023980901 | 2.786053335 | 0.009193545 | 2.58276103 | 0.03026923 |
| FKBP10 | -3.152606882 | 1.60E-20 | 2.780931431 | 0.002547739 | -4.734376967 | 2.57E-06 |
| TP53I11 | 5.217307139 | 0.002244824 | 2.777841128 | 0.005829933 | 2.611363198 | 0.008043779 |
| IGHV4-28 | 4.537758391 | 0.011660829 | 2.724686819 | 0.013761138 | 3.142843056 | 0.00734717 |
| IGHV1-69-2 | 8.944611905 | 2.92E-10 | 2.722215773 | 0.014068206 | 2.377449531 | 0.035659284 |
| FBN2 | -7.41634609 | 3.64E-47 | 2.704062694 | 0.015447292 | -3.382091512 | 0.005009943 |
| MIR155HG | 3.212149124 | 0.000478644 | 2.668971562 | 0.005379538 | 5.485398731 | 5.98E-08 |
| SERP2 | -1.64857001 | 0.026764923 | 2.658380036 | 0.002847139 | 2.105891751 | 0.038517289 |
| KRT34 | -1.961181248 | 0.020354158 | 2.607147701 | 0.018699631 | 3.027088682 | 0.010039876 |
| LCE2B | 4.200398445 | 0.021977997 | 2.597989365 | 0.01998235 | 2.38227671 | 0.046793323 |
| TRBV27 | 5.028556563 | 0.004873438 | 2.595607673 | 0.020094585 | 2.729266957 | 0.023884957 |
| RNASE7 | -3.393440066 | 3.39E-19 | 2.589246748 | 0.000342259 | 1.75051771 | 0.021631311 |
| TRBV6-6 | 4.984423437 | 0.005350882 | 2.577423649 | 0.020988783 | 2.719959089 | 0.02436702 |
| NECTIN3 | -3.982199507 | 2.94E-07 | 2.572957765 | 0.011534153 | 4.13594636 | 0.000237194 |
| IGHV3-20 | 8.002907353 | 4.34E-08 | 2.560269232 | 0.01000944 | 4.304064377 | 1.49E-05 |
| WNT5B | 4.251997282 | 0.019837354 | 2.552368858 | 0.021663441 | 2.956109041 | 0.01248635 |
| LPAR1 | -4.12561173 | 2.04E-40 | 2.544618359 | 0.003209321 | 3.032802763 | 0.000855264 |
| VASN | 4.413270083 | 0.015417468 | 2.5375272 | 0.022802959 | 2.878535576 | 0.016092982 |
| IGLC7 | 10.69413166 | 6.52E-13 | 2.526917458 | 0.012655288 | 2.979245132 | 0.002753386 |
| PLA2G4D | -2.441272129 | 1.39E-08 | 2.49894355 | 0.025233919 | 2.713986379 | 0.024545306 |
| PRKAA2 | -4.477316961 | 0.000191792 | 2.48752733 | 0.025489405 | 2.882573538 | 0.015300637 |
| KRTAP1-5 | -7.809249897 | 1.79E-08 | 2.48752733 | 0.025489405 | 2.882573538 | 0.015300637 |
| RAB3B | -4.398457005 | 1.40E-24 | 2.486674365 | 0.019603723 | 2.839761272 | 0.011031098 |
| RNF112 | 2.84780033 | 4.92E-10 | 2.341180722 | 0.035830927 | 2.749932769 | 0.021010064 |
| IGFL1 | 5.887485263 | 0.000394177 | 2.329150957 | 0.019395577 | 4.003253843 | 0.000328912 |
| HSPB3 | -4.744920582 | 1.81E-12 | 2.32304362 | 0.018483965 | 3.73758671 | 0.000178371 |
| CCL3 | 5.834651007 | 0.000399434 | 2.291722806 | 0.021266971 | -1.934725545 | 0.007614446 |
| LCE3E | 9.349026929 | 1.72E-11 | 2.285257952 | 0.040607021 | 2.706649698 | 0.023288655 |
| FOXF1 | -3.776165215 | 5.10E-23 | 2.069010579 | 0.040355966 | -3.180922325 | 0.001292897 |
| RNF150 | 3.974441802 | 0.032183128 | 2.06425863 | 0.046937104 | 2.757849726 | 0.010839298 |
| KIRREL3 | -6.033474119 | 5.81E-28 | 2.062629946 | 0.034982138 | 2.334758333 | 0.010979027 |
| RETN | -6.134083028 | 3.44E-30 | 2.032475635 | 0.047910121 | 3.7197288 | 0.001068669 |
| IGHV5-78 | 4.441128512 | 0.013756825 | 1.988708523 | 0.046462532 | 2.44035378 | 0.012370137 |
| TMEM155 | 4.967065441 | 0.00413931 | 1.97563981 | 0.049317537 | 2.051918411 | 0.023843365 |
| IGLV1-50 | 4.334390234 | 0.001358815 | 1.975422046 | 0.049277414 | 1.952554844 | 0.048652526 |
| SLIT2 | 3.309004336 | 8.31E-05 | 1.808258566 | 0.043874274 | 2.876184794 | 0.001539774 |
| RNU6-37P | -7.03207656 | 4.57E-06 | 1.734090253 | 0.036427659 | 1.431420838 | 0.002526907 |
| ARG2 | 2.912501321 | 2.35E-08 | 1.675275395 | 0.002367295 | -1.770945363 | 0.009146318 |
| OXTR | -2.542808512 | 1.21E-12 | 1.659808775 | 0.044318882 | 3.404231649 | 6.83E-05 |
| CCNJL | 2.101211409 | 4.43E-10 | 1.197964812 | 0.004254298 | -5.654261815 | 1.42E-08 |
| PCDHGC3 | -3.332682256 | 1.40E-36 | -0.792689164 | 0.021503391 | -1.452753492 | 0.000254432 |
| SAMD11 | 4.201262323 | 5.24E-07 | -1.088719863 | 0.038639332 | -0.620943334 | 0.019001849 |
| SV2A | -2.507037143 | 5.93E-06 | -1.131339953 | 0.001975425 | -1.582019079 | 0.01041076 |
| ARMCX4 | -1.916548706 | 1.93E-11 | -1.178646218 | 0.003978726 | 2.513427353 | 0.000673164 |
| INSL3 | 4.966449651 | 0.004144343 | -1.219275468 | 0.043117251 | -2.272653315 | 0.025805235 |
| NKX3-1 | 3.710869919 | 0.003139923 | -1.224987607 | 0.005564159 | -0.911407847 | 0.048813062 |
| VEGFC | -0.685666724 | 0.02437776 | -1.284511459 | 0.026626239 | -1.909435193 | 5.34E-05 |
| MMP17 | -3.469304041 | 2.92E-33 | -1.636430143 | 0.006338581 | -1.685824854 | 0.034835742 |
| VPS37D | -2.884158605 | 8.59E-09 | -1.637088858 | 0.003862583 | -1.607240968 | 0.045739786 |
| FARP1 | -3.107638251 | 1.44E-19 | -1.644909779 | 0.008378412 | -5.239331357 | 2.39E-06 |
| SDS | -4.82811788 | 2.64E-20 | -1.645316475 | 0.00055011 | -1.561943335 | 0.032920837 |
| DSEL | -3.645851455 | 1.82E-34 | -1.650983115 | 0.001145671 | -2.483416991 | 0.002275936 |
| GFRA1 | -3.702521415 | 2.25E-25 | -1.66294962 | 0.004084053 | -4.446789542 | 2.38E-08 |
| PLA2G2F | -4.590727693 | 5.51E-18 | -1.667904978 | 0.013252637 | -1.389667951 | 0.040094587 |
| KCNS1 | -3.502246767 | 1.48E-08 | -1.681219009 | 0.002709157 | -2.753037241 | 2.57E-05 |
| CCDC71L | -1.651846237 | 6.45E-05 | -1.685335238 | 0.009048004 | 2.894431994 | 0.001449798 |
| PLPPR1 | 3.869773711 | 0.000880368 | -1.725375625 | 0.005959599 | -1.626298829 | 0.01723183 |
| WFDC12 | -2.477886523 | 8.12E-13 | -1.737383866 | 0.011215906 | -2.929994482 | 0.000211126 |
| CBARP | -2.684057092 | 2.90E-17 | -1.746883234 | 0.029283091 | -4.039283481 | 0.000207324 |
| MSC | -2.830112986 | 7.59E-09 | -1.749679209 | 0.016885887 | -1.766512618 | 0.009036751 |
| DEFB104A | 4.413270083 | 0.015417468 | -1.752599735 | 5.95E-05 | -1.892388495 | 0.038086576 |
| PCDHGA12 | -4.027170095 | 4.79E-12 | -1.752940917 | 0.032972076 | -2.780035734 | 0.000656766 |
| KRT84 | 3.974967612 | 0.031757045 | -1.754195978 | 0.008568364 | -1.830279497 | 0.010379963 |
| PACSIN1 | -2.159534442 | 0.000845161 | -1.757931773 | 0.034533974 | -2.624305925 | 0.002903301 |
| LOXL2 | -3.985276542 | 1.48E-12 | -1.772484376 | 0.002890719 | -2.130743038 | 0.003890385 |
| SLED1 | -2.827123975 | 4.86E-05 | -1.775533541 | 0.034620828 | -2.125212592 | 0.020115689 |
| VCL | -2.590758459 | 1.55E-09 | -1.776637505 | 0.006389174 | -1.503490557 | 0.03653845 |
| CSF1 | -3.047799979 | 5.68E-12 | -1.785636667 | 0.002321573 | -4.111162979 | 0.00021873 |
| CPT1C | -2.841558844 | 3.58E-13 | -1.801346409 | 0.016428936 | -4.537200081 | 1.49E-05 |
| AIFM2 | -2.686681748 | 8.23E-34 | -1.802571196 | 0.004829601 | -4.039483767 | 0.000106284 |
| GDA | 5.221590906 | 1.45E-07 | -1.810395194 | 0.029724929 | -3.533228812 | 0.000186697 |
| AMOT | -2.824594117 | 4.08E-15 | -1.811627226 | 0.001497678 | -1.823141044 | 0.021087045 |
| BCAT1 | -2.841355669 | 3.58E-11 | -1.822208608 | 0.01193087 | -1.97243467 | 0.008665646 |
| STAC2 | -3.163329927 | 7.91E-27 | -2.00243164 | 0.007044191 | -1.686314221 | 0.048967147 |
| CACNA1C | -3.071630918 | 9.26E-19 | -2.006702722 | 0.00095158 | 3.724835876 | 0.000162518 |
| SMURF2 | -2.869606453 | 3.62E-30 | -2.013969752 | 0.004766508 | -1.627381924 | 0.031901089 |
| CRLF1 | 2.716105995 | 0.000156338 | -2.015300262 | 0.034547774 | -1.406512481 | 0.013644684 |
| FOXD1 | -4.580229102 | 3.62E-26 | -2.276596656 | 0.002051144 | 2.476172559 | 0.013377 |
| FGF5 | -11.08976062 | 1.97E-32 | -2.276892841 | 0.001265243 | 2.008468353 | 0.017779745 |
| P3H3 | 3.638018861 | 1.69E-05 | -2.506763425 | 0.006468149 | -2.95103824 | 0.002991783 |
| TIMP3 | -3.194554359 | 7.67E-08 | -2.713374951 | 0.000841994 | -1.888771289 | 0.010126268 |
| TGFB2 | -3.984464839 | 2.25E-14 | -2.793785948 | 3.00E-06 | -2.040202686 | 0.008709673 |
| BAG2 | -3.662837675 | 5.97E-29 | -2.878117734 | 0.000470939 | -4.779871406 | 3.84E-06 |
| LOR | 9.69776419 | 2.24E-12 | -2.901583206 | 0.000908513 | -2.254405317 | 0.020071632 |
| SIX1 | -3.620434215 | 6.64E-10 | -2.903690356 | 0.001323622 | -2.199271366 | 0.023855683 |
| NACAD | -3.396948466 | 3.31E-11 | -2.917889526 | 0.00271393 | -2.856268287 | 0.00652351 |
| CSDC2 | -4.428185614 | 1.80E-07 | -2.92105952 | 0.002740483 | 3.308496183 | 0.002029141 |
| ITGA11 | -2.876720602 | 0.000644502 | -2.923013192 | 0.000248841 | -3.297847811 | 1.92E-05 |
| FRMPD1 | 5.091341292 | 0.0031074 | -3.856324576 | 6.37E-05 | -1.867361068 | 0.021031712 |
| ITGA8 | -5.57573838 | 1.05E-58 | -3.88881682 | 9.54E-06 | -3.314266952 | 0.000793415 |
| REEP2 | -4.065024319 | 1.18E-23 | -3.919571385 | 0.000119211 | -3.705907023 | 0.00016836 |
| MEIS3 | -3.125262552 | 4.84E-20 | -4.030856832 | 0.000100858 | -3.294985856 | 0.003649202 |
| NQO1 | 5.531232321 | 0.000920952 | -4.044129481 | 1.10E-05 | -3.579393277 | 0.000214226 |
| PTGS1 | -3.911339494 | 7.70E-27 | -5.1508104 | 1.99E-07 | -5.03641669 | 5.22E-06 |
| GREM1 | -7.445816381 | 1.01E-62 | -5.25809095 | 5.22E-07 | -1.980187846 | 2.60E-08 |
| NLGN1 | -1.92715158 | 3.13E-12 | -5.418065735 | 4.41E-08 | -4.586697853 | 7.79E-05 |

Figure S1.


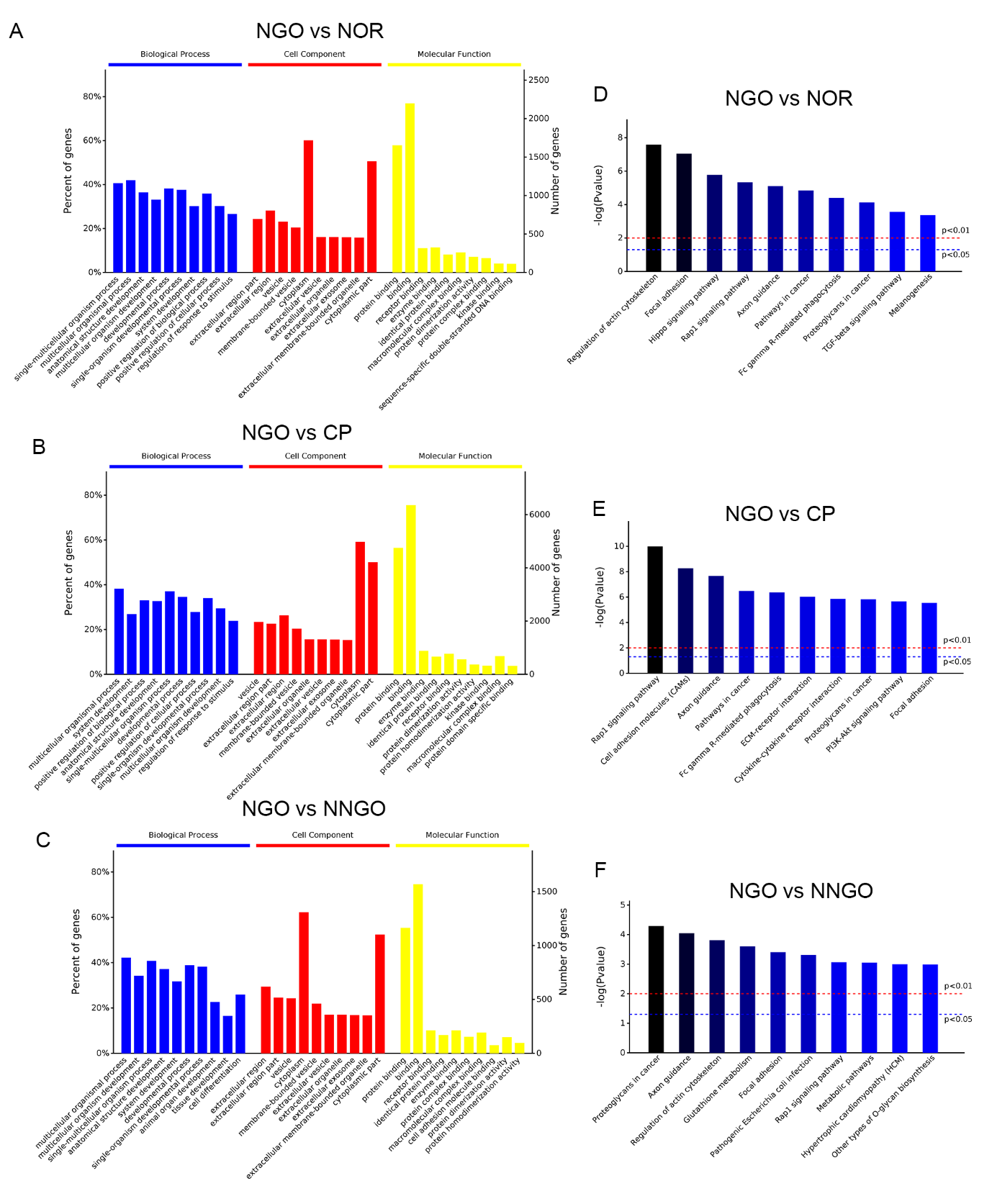


Figure S2.


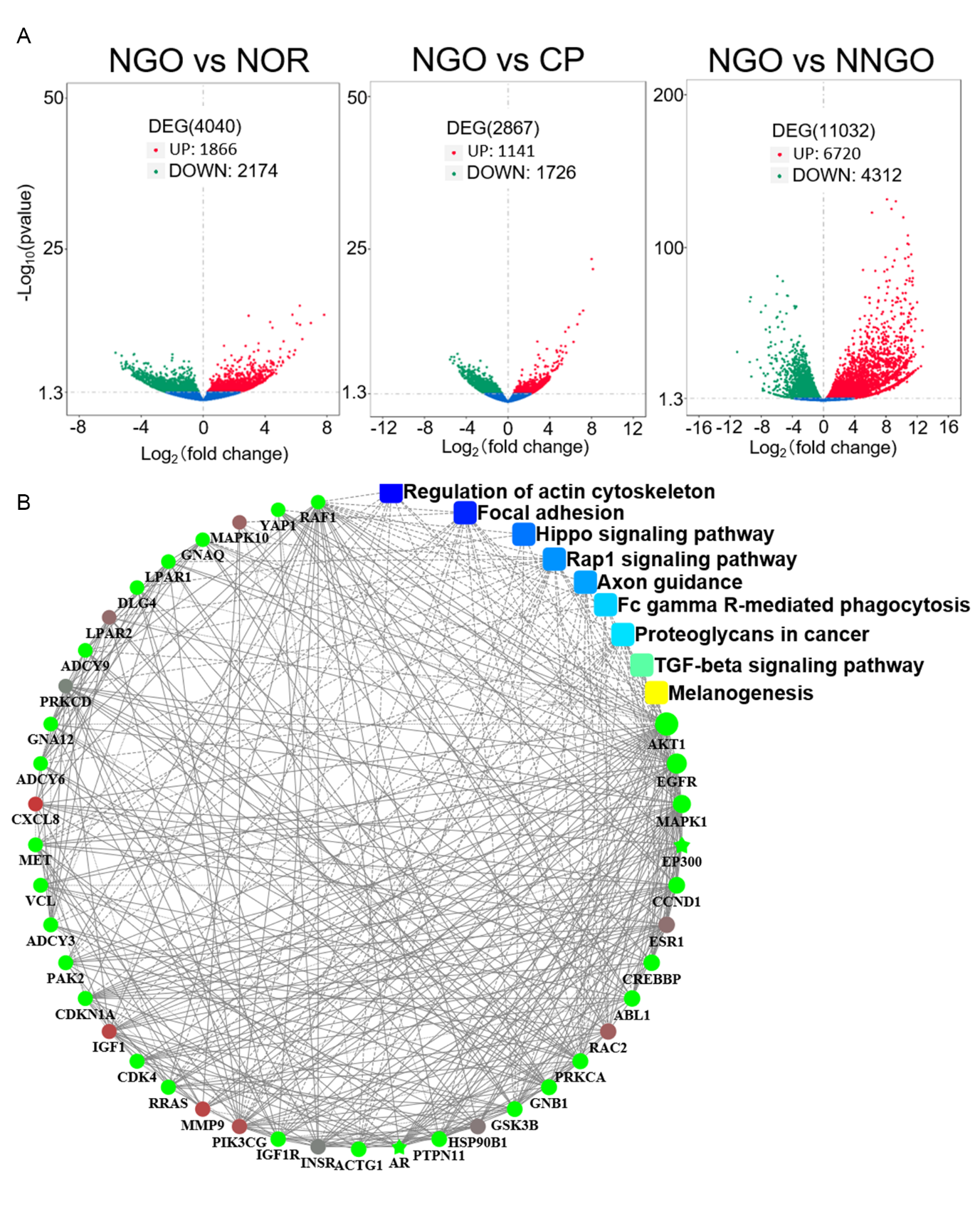

Supplement: Supplementary Materials — Table S1: list of the main overlapping genes. Figure S1: (A–C) the GO analysis of DEGs in NGO group versus NOR, CP, and NNGO, respectively. (D–F) KEGG analysis of those groups. Figure S2: RNA-Seq analysis of NGO vs. other groups. (A) Volcano plot to identify the fold changes and statistical significance. (B) Protein–protein interaction network of the differentially expressed genes (DEGs). [file 6128341.f1.docx]
